# Identification and discrimination of *Theileria annulata* by polymerase chain reaction-restriction fragment length polymorphism

**DOI:** 10.14202/vetworld.2022.925-929

**Published:** 2022-04-14

**Authors:** Abdelfattah Selim, Hanem Khater

**Affiliations:** 1 Department of Animal Medicine (Infectious Diseases), Faculty of Veterinary Medicine, Benha University, Toukh 13736, Egypt; 2 Department of Parasitology, Faculty of Veterinary Medicine, Benha University, Toukh 13736, Egypt

**Keywords:** cattle, Egypt, genotype, polymerase chain reaction-restriction fragment length polymorphism, *Theileria annulata*

## Abstract

**Background and Aim:**

*Theileria annulata* infection is a tick-borne disease affecting ruminants in the tropical and subtropical regions causing severe economic losses. This study aimed to characterize circulating *T. annulata* isolates from four governorates (administrative districts) north and south of Egypt using polymerase chain reaction (PCR)-restriction fragment length polymorphism.

**Materials and Methods:**

Fifty samples were collected from the four governorates of Egypt and were examined by a PCR assay based on the heat shock protein 70 gene. The amplified product was subsequently digested using two restriction enzymes, Taq I and Alu I, to determine which pattern of *T. annulata* strains was involved.

**Results:**

The findings revealed that one distinct pattern was observed for *T. annulata* isolates in the northern governorates and another one in the southern governorates. The Taq I enzyme produced three fragments (100, 175, and 270 bp), and the Alu I enzyme produced four fragments (60, 90, 125, and 270 bp).

**Conclusion:**

This study determined the presence of two distinct circulating genotypes of *T. annulata* among cattle in Egypt based on PCR-RFLP using the HSP 70 gene. More studies are needed in different parts of the country to investigate the virulence and strain variance of *T. annulata* in cattle.

## Introduction

Bovine theileriosis is a tick-borne disease, caused by an intracellular protozoan parasite *Theileria annulata* [1], which is transmitted by ticks belonging to *Hyalomma* species and causes significant economic losses to the dairy and cattle industries [2-4]. Theileriosis commonly spreads among cattle in the tropical and subtropical regions and is prevalent worldwide. It spreads from Asia through the Middle East [5] to North Africa [6] and South Europe [7].

The clinical features of tropical theileriosis are determined by the damaging effects of the parasite on the host lymphatic tissues and immune system [8-10]. The most common clinical signs of theileriosis are fever, anorexia, diarrhea, pre-scapular and pre-femoral lymph node enlargement, respiratory distress, jaundice or anemic mucous membrane, and corneal opacity of the eye [3,11].

According to a previous study conducted by El-Dakhly *et al*. [12], exotic and crossbred animals are more vulnerable to *T. annulata* infection than local Egyptian cattle breeds.A decreased infection rate in local cattle breeds could be attributable to a delicate balance between infection and the immune system of the animal [13]. Numerous isolates and strains of *T. annulata* found in an endemic area have varying levels of virulence in susceptible populations. As a result, the parasite isolate involved has a significant impact on the pathogenesis of bovine theileriosis [14,15]. Sequencing of the relevant genes is one of the primary methods for detecting parasite strain variations; however, it is not cost-effective [16-18].

The use of selective enzymes for restriction digestion of polymerase chain reaction (PCR) allows monitoring of many samples. PCR-restriction fragment length polymorphism (PCR-RFLP) was frequently used for the specific detection and identification of *T. annulata* based on specific target genes such as the tumor-associated macrophages (TAMs)-1 gene [19], SmI-2 gene [20], b-tubulin gene [15], and heat shock protein (HSP) 70 gene [21]. HSPs are the most conserved proteins in organisms and play a critical role in evoking the host’s reaction to stress [22]. They are particularly important in parasites involving vectors because when a parasite is transmitted from a poikilothermic invertebrate vector to a homeothermic vertebrate host, the parasite’s environment is drastically altered [23]. The HSP gene plays an important role in parasite survival and/or development within the host by adapting to diverse stress stimuli in general and temperature fluctuations [21,24].

The study aimed to characterize field isolates of *T. annulata* using PCR-RFLP targeting the HSP 70 gene with Taq I and Alu I restriction enzymes.

## Materials and Methods

### Ethical approval

The study was approved by the Ethical Research Committee, Faculty of Veterinary Medicine, Benha University, Egypt.

### Study period and location

The study was conducted from May 2020 to January 2021 in two governorates (Alexandria and Menofia) situated in North Egypt and the other two governorates (Minya and Beni Suef) situated in South Egypt. These governorates have a hot desert climate, which the Köppen-Geiger classification classifies as BWh. The weather is characterized by high humidity (40%), warm temperature (20-35°C), and low rainfall (100-200mm), which favors for multiplication of ticks vectors, particularly *Rhipicephalus annulatus* and *Hyalomma anatolicum* that implicated in bovine theileriosis spreading.

### Sampling

**Figure-1:**
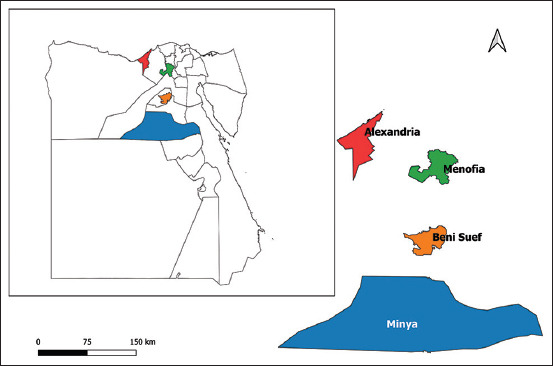
Map for the study area [Map generated by QGIS software].

A total of 50 blood samples were collected from clinically infected cattle representing four governorates (administrative districts) (Figure-1). Twenty blood samples were collected from Minya and Beni Suef and 30 blood samples were from Alexandria and Menofia. Blood samples (5 mL) were collected from infected cattle during parasitemia and put into collecting tubes with ethylenediaminetetraacetic acid. All samples were examined using microscopy and a PCR assay targeting the TAMs1 gene to confirm *T. annulata* positivity.

### DNA extraction and PCR amplification

The parasite DNA was extracted from blood samples using a QIAamp^®^ DNA mini kit (Qiagen GmbH, Hilden, Germany), according to the manufacturer’s protocol. The purified DNA was preserved at −20°C until PCR was performed.

The PCR assay was performed using a specific set of primers targeting the HSP 70 gene of *T. annulata* to amplify a 275 bp fragment as previously described [21]. The forward primer 5’TGTCAAGGAGGCCTCAAA TTA3’ and the reverse primer 5’TTTGACTTTGAATAGGCTGCC3’ were used. The thermal condition started by initial denaturation at 95°C for 2 min followed by 35 cycles of denaturation at 94°C for 30 s, annealing at 55°C for 45 s, and elongation at 72°C for 30 s. Then, there was a final extension for 10 min at 72°C. The amplified products were separated by electrophoresis on a 2% agarose gel and visualized with an ultraviolet (UV) transilluminator.

### Restriction enzyme analysis

Following PCR amplification, Taq I and Alu I restriction enzymes (10U, Fast Digest enzymes) were used to digest the generated PCR products. Taq I enzyme restriction digestion was performed at 65°C for 5 min, while Alu I enzyme digestion was performed at 37°C for 15 min. The digested products were separated by electrophoresis on a 2% agarose gel and visualized using a UV transilluminator.

## Results

### Molecular findings

**Figure-2:**
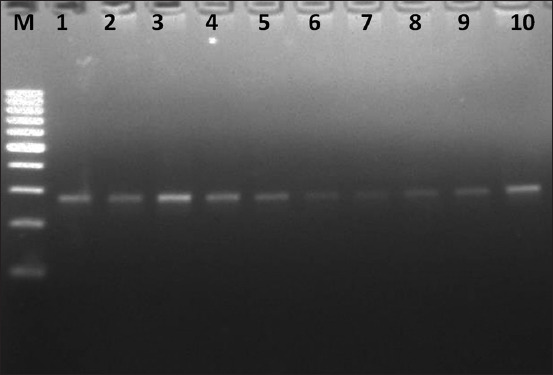
Agarose electrophoresis for amplified products. Lane M: 100 bp marker, lanes 1-10: Positive samples (275 bp).

The extracted DNA of *T. annulata* was examined by a PCR assay targeting the HSP 70 gene for 50 samples representing the north and south of Egypt. All samples were positive and produced a detectable 275 bp band (Figure-2).

### PCR-RFLP analysis

**Figure-3:**
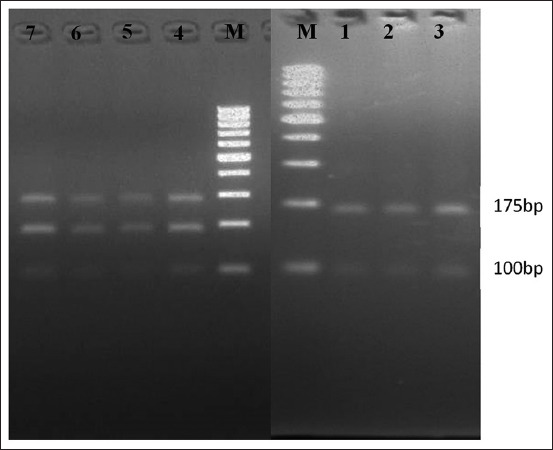
Restriction fragment length polymorphism (RFLP) analysis for polymerase chain reaction (PCR) products. Lane M: 100 bp marker, lanes 1-3: RFLP analysis for PCR product digested by TaqI (samples from North Egypt), and lanes 4-7: RFLP analysis for PCR product digested by TaqI (samples from South Egypt).

**Figure-4:**
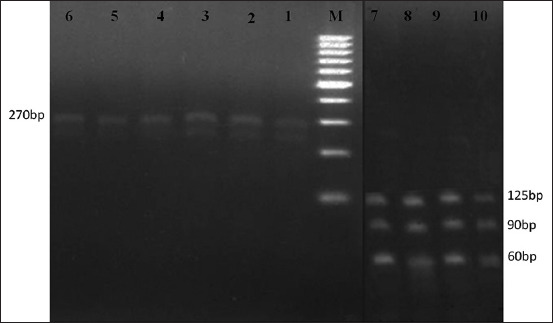
Restriction fragment length polymorphism digestion for AluI enzyme. Lane M: 100 bp marker, lanes 1-6: Identified restriction digestion products of AluI (270 bp) for northern samples, and lanes 7-10: Identified restriction digestion products (60, 90, and 125 bp) for southern samples.

The TaqI and AluI restriction enzymes were used to digest the amplified 275 bp product of the samples to detect the variations in the nucleotide sequences of the HSP 70 gene. The PCR-RFLP pattern confirmed the presence of two different genotypes for circulating *T. annulata*. The results revealed that TaqI digested all samples and produced three identified fragments (100, 175, and 270 bp) in the samples from the Minya and Beni Suef governorates (20/50, 40%). In contrast, it gave two fragments (100 and 175 bp) for the samples collected from the Alexandria and Menofia governorates (30/50, 60%) (Figure-3). Moreover, all PCR products were digested with AluI. Three 60, 90, and 125 bp fragments were produced with samples collected from Alexandria and Menofia (30/50, 60%). In contrast, only one fragment (270 bp) was produced from the samples collected from the Minya and Beni Suef governorates (20/50, 40%) (Figure-4).

## Discussion

Theileriosis is a common tick-borne disease that affects ruminants and is endemic to several parts of Egypt [25-27]. In the present work, cattle with clinical theileriosis were analyzed molecularly to determine the species implicated in bovine *Theileria* infection in North and South Egypt.

RFLP is a widely used method for distinguishing parasites based on patterns created by the cleavage of their DNA or a certain gene. Dissimilarities in cleavage/digestion patterns are frequently used to distinguish closely related species and strains [27-29]. The PCR-RFLP method effectively identifies genotypic differences in *T. annulata* isolates quickly [21,30]. Genes such as Sml-2 [20], TAMs [19], 18S rRNA [19], and HSP 70 [24] genes are frequently used to identify *T. annulata* strain differences in vectors or vertebrate hosts.

The HSP 70 gene was used in this investigation for restriction digestion with TaqI and AluI enzymes. The TaqI and AluI enzymes digested PCR products of all *T. annulata* isolates representing two different PCR-RFLP patterns. In contrast, PCR-RFLP based on the HSP 70 gene with TaqI and Alu enzymes was used for the characterization of *T. annulata* from the blood of infected cattle and ticks affecting livestock in Iran and revealed only one pattern [19,24]. However, the results from the present study are similar to those of Akbari *et al*. [20]. They found two genotypes for *T. annulata* among Iranian cattle using PCR-RFLP based on the Sml-2 gene with TaqI and AluI enzymes.

Using PCR-RFLP based on the TAMs 1 gene with the Rsa I enzyme revealed four distinct genotypes for *T. annulata* in both cattle and tick vectors in Iran [19,31]. The presence of a polymorphism in the TAMs 1 gene can explain the detection of a significant number of circulatory genotypes utilizing the TAMs 1 gene [32].

A further novel finding is that all isolates could be digested with both restriction enzymes, yielding two distinct restriction fragments, indicating strain diversity between *T. annulata* species circulate among cattle in North and South Egypt. However, the results confirmed that only one genotype is present in the north and another is in the south of Egypt [12,33].

## Conclusion

This study determined the presence of two distinct circulating genotypes of *T. annulata* among cattle in Egypt based on PCR-RFLP using the HSP 70 gene. More studies are needed in different parts of the country to investigate the virulence and strain variance of *T. annulata* in cattle. There is a potential that mass vaccination and/or an effective diagnostic tool will fail due to the presence of a large number of circulating genotypes, resulting in a lot of strain variation.

## Authors’ Contributions

AS and HK: Conceptualization, methodology, formal analysis, investigation, resources, data curation, and writing – original draft preparation. AS and HK: Writing – review and editing. AS and HK: Project administration. AS and HK: Funding acquisition. All authors have read and approved the final manuscript.
